# Semantic Description of Data Mining Datasets: An Ontology-Based Annotation Schema

**DOI:** 10.1007/978-3-030-61527-7_10

**Published:** 2020-09-19

**Authors:** Ana Kostovska, Sašo Džeroski, Panče Panov

**Affiliations:** 8 University of Bari Aldo Moro, Bari, Italy; 9 Aristotle University of Thessaloniki, Thessaloniki, Greece; 10 Open University of Cyprus, Nicosia, Cyprus; 11 Dalhousie University, Halifax, NS Canada; 12 Department of Knowledge Technologies, Jožef Stefan Institute, Ljubljana, Slovenia; 13 Jožef Stefan International Postgraduate School, Ljubljana, Slovenia

**Keywords:** Data mining, Datasets, Knowledge representation, Semantic annotation, Ontology

## Abstract

With the pervasiveness of data mining (DM) in many areas of our society, the management of digital data, readily available for analysis, has become increasingly important. Consequently, nearly all community accepted guidelines and principles (e.g. FAIR and TRUST) for publishing such data in the digital ecosystem, stress the importance of semantic data enhancement. Having rich semantic annotation of DM datasets would support the data mining process at various choice points, such as data understanding, automatic identification of the analysis task, and reasoning over the obtained results. In this paper, we report on the developments of an ontology-based annotation schema for semantic description of DM datasets. The annotation schema combines three different aspects of semantic annotation, i.e., annotation of provenance, data mining specific, and domain-specific information. We demonstrate the utility of these annotations in two use cases: semantic annotation of remote sensing data and data about neurodegenerative diseases.

## Introduction

Recently, the success of Data Mining (DM) and Machine Learning (ML) in a broad range of applications has led to a growing demand for ML systems. However, this success heavily relies on the ML expertise of the practitioners, and on the quality of the analyzed data, both of which are in short supply. One potential solution for overcoming the shortage of expertise is to develop more intelligent data analysis systems, that will assist domain practitioners in the construction of analysis pipelines and the interpretation of results. Such an intelligent DM system would we able to reason over distributed heterogeneous data and knowledge bases, automatically define the learning task, recommend the most suitable algorithms for the task at hand, and correctly interpret the induced predictive models [17, 18].

The first step towards the development of such systems is the improvement of data management and data understanding. Research data must be enriched with formal and logical descriptors that capture the characteristics of the data relevant for the task of automation of the data analysis process. Additionally, these descriptors have the potential to significantly improve interdisciplinary research by helping ML practitioners better understand the data originating from the application domains, as well as easily incorporate domain knowledge in the process of analysis. Formal descriptors, when published on the Web, can also improve the accessibility and reusability of scientific data.

Many academic institutions have recognized the importance of effective management of scientific data, making it their central mission. For example, the FAIR (Findable, Accessible, Interoperable, and Reusable) principles [26] are a set of guiding principles that have been introduced to support and promote proper data management and stewardship. In that context, data must be discoverable and it should be semantically annotated with rich metadata. The metadata should always be accessible by standardized communication protocols. The data and the metadata have to be interoperable with external data from the same domain. Finally, both data and metadata should be released with provenance details so that the data can be easily replicated and reused.

Another set of principles that builds upon FAIR data are the TRUST principles [13]. The TRUST principles go a level higher by focusing on data repositories and providing them with guidance to demonstrate Transparency, Responsibility, User focus, Sustainability, and Technology (TRUST).

At the core of both principles lies the semantic enrichment of research data. Semantic annotation of data, as a powerful technique, has attracted attention in many domains. Unfortunately, semantic annotation of DM and ML datasets is still in the early phases of development. To the best of our knowledge, there are no semantic dataset repositories from the general area of data science that completely adhere to the FAIR and TRUST principles.

In this paper, we report on the development of an ontology-based annotation schema for semantic annotation of DM datasets. Our main objective is to provide a rich vocabulary for data annotation, that will serve as a basis for the construction of a dataset repository that closely follows the FAIR and TRUST principles. The annotation schema we proposed includes three different types of information: provenance, DM-specific, and domain-specific. The provenance information improves the transparency and reusability of data. The DM-specific information provides means for reasoning over the analyzed data and helps (in a semi-automatic way) in the construction of the DM workflows (or pipelines). The domain-specific information helps to bridge the gap between ML practitioners and domain experts, as well as to improve cross-domain research. Finally, we demonstrate the utility of domain-specific annotations in two use cases from the domains of neurodegenerative diseases and Earth Observation (EO), respectively.

## Background and Related Work

In the context of computer science, ontologies are “an explicit formal specifications of the concepts and the relations among them that can exist in a given domain” [9]. In other words, they provide the basis for an unambiguous, logically consistent, and formal representation of knowledge. It is important to note that, the logical component of ontologies allows knowledge to be shared meaningfully both at machine and human level. Also, an immediate consequence of having formal ontologies based on logic is that they can be used in a variety of reasoning tasks, as well as in the inference of new knowledge. The benefits of having ontology-based knowledge representations have been demonstrated in many data- and knowledge-driven applications. The research areas that retained most attention and contributed the most to the technological breakthrough of ontologies are bioinformatics and biomedicine. For example, the Open Biological and Biomedical Ontology (OBO) Foundry [21] is a collective of ontology developers that have developed and maintain over 100 publicly-available ontologies related to the life sciences. When it comes to the process of ontology engineering, the OBO Foundry has played a key role, as they have proposed ontology design principles that promote open, orthogonal, and strictly-scoped ontologies with collaborative development. These principles have further widened the use of ontologies across different fields of science.

In the area of DM and ML, a large body of research has focused on the development of ontologies, vocabularies and schemas that cover different aspects of the domain. Examples of such resources include the Data Mining OPtimization Ontology (DMOP) [11], Exposé [24], MEX vocabulary [8], and the ML schema [7]. DMOP has been designed to support automation at various choice points of the DM process. The Exposé ontology provides the vocabulary needed for a detailed description of machine learning experiments. MEX represents a lightweight interchange format for ML experiments. ML Schema represents an effort to unify the representation of machine learning entities.

The OntoDM suite of ontologies is of particular interest, as this paper extends its line of work. OntoDM includes three different ontologies: OntoDM-core, OntoDM-KDD, and OntoDT. OntoDM-core [17] is an ontology of core data mining entities, such as dataset, DM task, generalizations, DM algorithms, implementations of algorithms, and DM software. OntoDM-KDD [16] is an ontology for representing the process of knowledge discovery following the CRISP-DM methodology [5]. OntoDT [18] is a generic ontology for the representation of knowledge about datatypes.

Another type of information related to DM datasets that is important to be formally represented is the provenance information. Provenance information refers to the kind of information that describes the origin of a resource (in our case a dataset), i.e., who created the resource, when was it published, and what is its usage license. Provenance information is valuable when it comes to deciding whether a specific resource can be trusted. This extra information also helps the users better understand it, easily cite and reuse the resource for their purposes. For the computers to make use of the provenance information, it has to be given explicitly, and it has to be based on common provenance vocabularies, such as the Dublin Core vocabulary [25], the PROV ontology [2], Data Catalog Vocabulary [1], or Schema.org [3].

## Semantic Description of DM Datasets

To semantically describe a DM dataset, we consider three different types of vocabularies/ontologies: (1) vocabularies for annotation of provenance information, such as title, description, license, and format; (2) ontologies for annotation of datasets with DM-specific characteristics, i.e., data mining task, datatypes, and dataset specification; and (3) ontologies for annotation of domain-specific knowledge that helps to contextualize the data originating from a given domain.

In this section, we discuss the first two aspects of the semantic enrichment of datasets. We describe the Schema.org vocabulary, which we reuse for the purpose of annotation of the dataset’s provenance details. Also, we outline the main characteristics of the OntoDT and OntoDM-core ontologies and we further extend their structure with terms essential for semantic description from a DM perspective. In Sect. 4 we discuss the annotation of domain specific knowledge through examples from two different domains.

### Provenance Information Annotation

To annotate DM datasets with provenance information, we have chosen the Schema.org vocabulary, one of the most widely used vocabularies that provides descriptors for provenance information in a structured manner. When annotating the datasets, we usually use a subset of the list of provided descriptors as the complete provenance information is not always available.

**Fig. 1.**
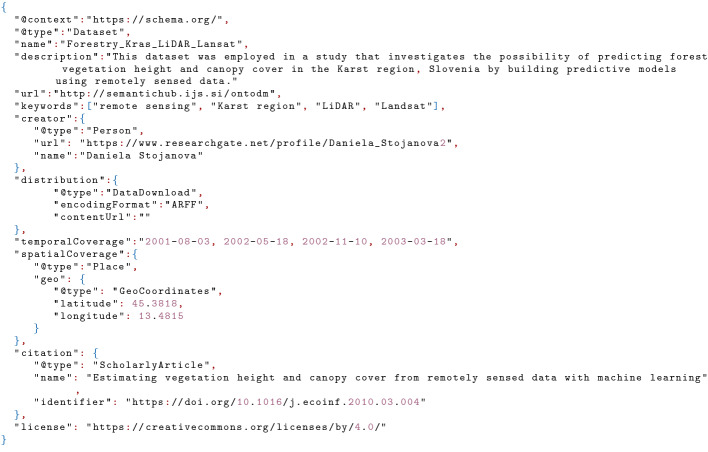
An example provenance information annotation for the Forestry Kras Lidar/Landsat dataset [23] using the Schema.org vocabulary.

Figure 1 depicts an example annotation of provenance information in JSON-LD format^1^. For this example, we used a dataset from the domain of Earth Observation (EO), named *Forestry_Kras_LiDAR_Landsat*. The dataset was used in a study that investigates the possibility of predicting forest vegetation height and canopy cover in the Karst region in Slovenia by building predictive models using EO data [23]. For semantic annotation of provenance information for this dataset, we used several terms from Schema.org, such as name, description, URL, keywords, creator, distribution, temporal and spatial coverage, citation, and license.

### Data Mining Specific Annotations

The second type of annotation considers explicit specification of dataset characteristics from a DM perspective, e.g., the format of the data, the type of learning task, and the features’ datatypes. Data used in the process of DM can take various forms, but the standard one assumes that there is a set of objects of interest described with features (or attributes). In that sense, the term data example, or (more commonly) data instance, refers to a tuple of feature values corresponding to an observed object.

The features are formally typed, meaning that each of them has a designated datatype. In general, there are many different datatypes such as boolean, real, discrete datatype, to name a few. Having standardized datatype information at disposal can enable the development of knowledge-based systems that automate parts of data analysis workflows, e.g., assist DM practitioners in choosing a suitable learning algorithm for the data at hand.

Data examples in DM can be described with different characteristics, which can lead to treating the data in radically different ways. We identified four different (orthogonal) characteristics that we believe are important to be represented appropriately. These include (1) the availability of data examples, (2) the existence of missing values, (3) the mode of learning, and (4) the type of target in the case of (semi-)supervised learning tasks.

**Extending the OntoDT and OntoDM-core Ontologies.** While the OntoDT and OntoDM-core ontologies offer a rich vocabulary for the annotation of DM datasets, they do not cover all of the above aspects. Thus, we extended OntoDT with new DM-specific datatypes and provided an updated datatype taxonomy that allows us to properly describe DM datasets. The proposed taxonomy of datatypes was then used as a basis for the update of the taxonomies of DM tasks and data specification, which are part of the OntoDM-core ontology. The extended OntoDT and OntoDM-core ontologies are available at https://w3id.org/OntoDT-extended and https://w3id.org/OntoDM-core-extended, respectively.

**Availability of the Data Examples.** Based on the availability of the data examples, we distinguish between two types of data, i.e., batch data (or datasets) and online data (or data streams). The batch setting is the more traditional approach where large volumes of data are collected over a longer period. On the other hand, online data refers to the type of data that is continuously being generated by heterogeneous data sources.

**Fig. 2.**
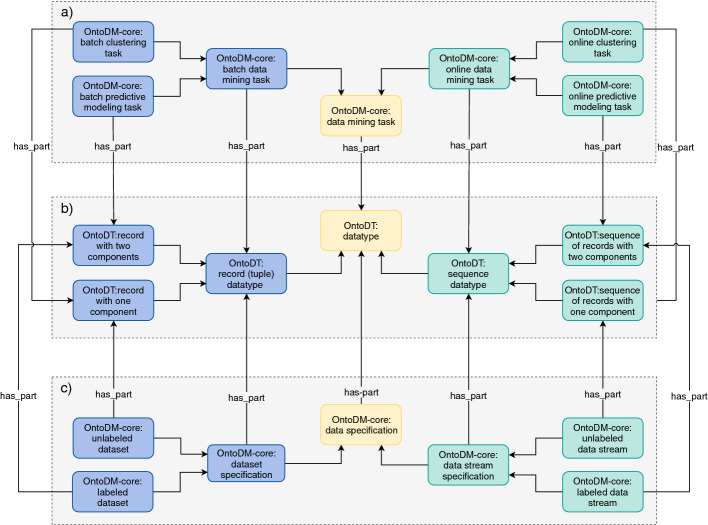
Top level overview of the taxonomies of data mining tasks, datatypes and data specifications for the batch setting (right-hand side) and online setting (left-hand side).

The availability of data examples is the first dimension we considered when we updated the taxonomies of core classes of OntoDT and OntoDM-core. In Fig. 2, we depict the top-level classes of the taxonomies of datatypes, data mining tasks, and data specifications. At the second level, we have the corresponding classes that represent the specifications of the availability of data examples. For instance, the *OntoDT: record(tuple) datatype* and *OntoDT: sequence datatype* classes refer to the datatypes of data examples in batch and online mode, respectively.

**Fig. 3.**
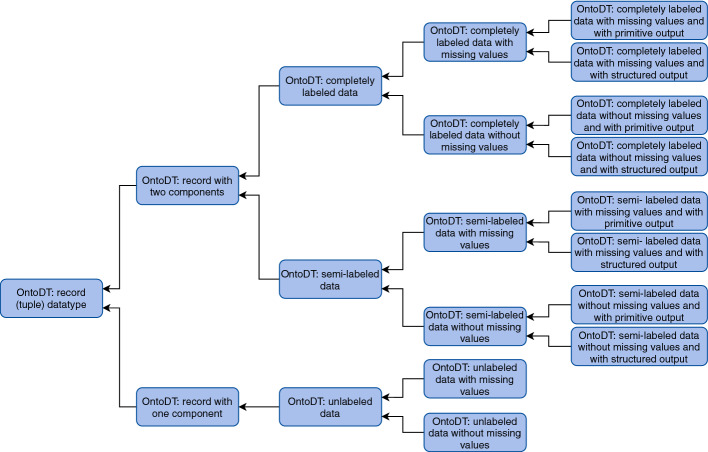
A part of the OntoDT datatype taxonomy.

**Type of Learning.** According to the type of learning, DM learning methods can be categorized into three groups, i.e., unsupervised, supervised, and semi-supervised learning. The key difference between them is the completeness of the data they use for training. Unsupervised learning makes use of unlabeled data examples that are only composed of descriptive features. Supervised learning, in contrast to unsupervised learning, uses labeled data that, apart from the descriptive features, has some special feature of interest usually referred to as target. Finally, in semi-supervised learning, we have learning from both labeled and unlabeled data examples.

In the updated taxonomies, we modeled this characteristic at the second level. Hence, for both batch and online learning, we defined classes that specify information about the type of learning (see Fig. 2). If we take the taxonomy of data types as an example, in the batch learning scenario the *OntoDT: record (tuple) datatype* class further resolves into two classes: *OntoOT: record with one component* and *OntoDT: record with two components*. *OntoOT: record with one component* class represents the datatype of data examples used in unsupervised batch learning mode, where there is only one descriptive component that aggregates the descriptive features of the data example. The *OntoDT: record with two components* class represents the datatype of data examples that have one descriptive and one target component and are used in either supervised or semi-supervised learning. Figure 3 illustrates in greater detail the taxonomy of data types and the four dimensions that it is based on. Finally, the taxonomies of tasks and dataset specifications are designed similarly following the same principles (see Fig. 2).

**Missing Values.** Another property we consider when describing data examples, which is important for DM algorithms, is the existence of missing values (see Fig. 3) since some DM algorithms cannot function properly in the presence of missing values. We say that one data example has missing values when there is no recorded value for at least one descriptive feature. This is different from having missing values in the target space, which, as we discussed above, leads to semi-labeled data. Missing values affect the data quality; thus they must be handled accordingly by the DM algorithms.

**Fig. 4.**
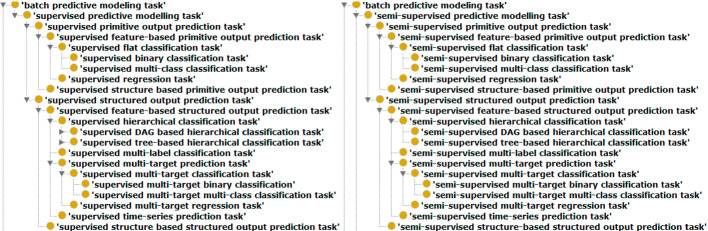
A Protégé snapshot of the taxonomy of supervised and semi-supervised batch predictive modeling tasks.

**Fig. 5.**
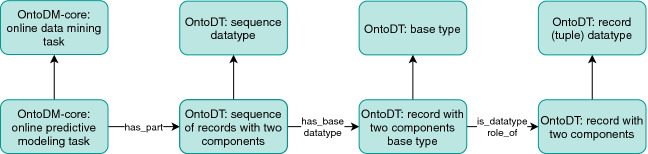
An example of modeling online data mining tasks with the corresponding datatypes from OntoDT.

**Type of Target.** In the case of (semi-)supervised learning, data examples can become even more complex as the target/output itself can have a complex structure. Based on the type of target we have primitive and structured output prediction tasks. Primitive output prediction tasks predict a single target, as in classification (a discrete value) and regression (a real value). In the case of structured output prediction tasks, there is more than one target that has to be predicted. Examples of such tasks are multi-target regression, multi-label classification, and hierarchical multi-label classification. Figure 4 presents the complete taxonomy of supervised and semi-supervised predictive modeling tasks.

Concerning the (semi-)supervised online predictive modeling tasks, the base datatypes of the target can be the same as the target datatypes in the batch predictive modeling tasks. Figure 5 illustrates how this is achieved in the OntoDT and OntoDM-core ontologies. For instance, *OntoDM-core: online predictive modeling task* class is related with the *OntoDT: sequence of records with two components* class. Sequence datatypes have a base datatype, in this example, it is the *OntoDT: record with two components base type*, which has the datatype role of *OntoDT:record of two components*. Note that *OntoDT:record of two components* is the same class used for the representation of the data examples’ datatype in the batch predictive learning mode.

### Example Annotations of DM Datasets

Using this annotation schema, we have annotated 496 DM datasets in total, all containing data from different application domains. The generated semantic annotations are publicly available in RDF format and can be queried via the Jena Fuseki server^2^.

**Fig. 6.**
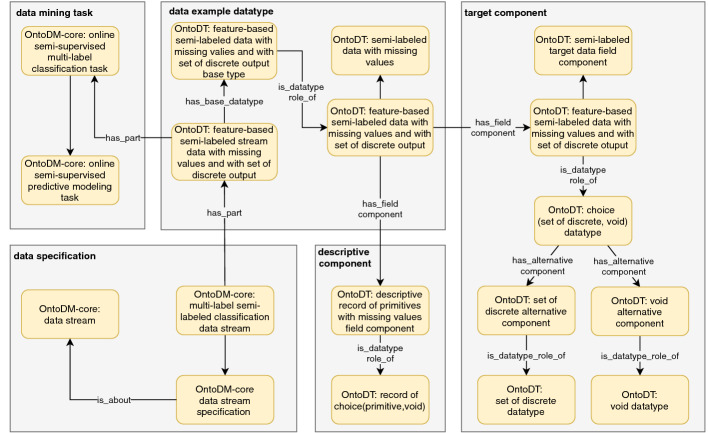
An example of an annotation schema for data streams applicable to semi-supervised multi-label classification.

After describing the four characteristics that govern the modeling of the taxonomies of datatypes, data specification, and tasks, we provide an illustrative example that shows how we can combine them in a single annotation schema for the purpose of semantic annotation of DM datasets. Namely, Fig. 6 depicts the classes needed for annotation of a data stream with missing values applicable to the learning task of semi-supervised multi-label classification.

To represent the datatype of the data examples, we use the *OntoDT:feature-based semi-labeled stream data with missing values and with a set of discrete output* class. This class is connected via the has-part relation with the classes that represent the corresponding data mining task and data specification defined in the OntoDM-core ontology, i.e., *OntoDM-core: online semi-supervised multi-label classification task* and *OntoDM-core: multi-label semi-labeled classification data stream*. The annotation schema for data streams includes also a specification of a base datatype. Next, we have the classes used for describing the datatypes of the descriptive and target component. On the descriptive side, some of the examples can have missing values, thus, we use a record/tuple of choice (primitive, void) datatypes. For the target component, we have two alternatives, one of which is a discrete datatype used for annotation of labeled examples, and the other is a void datatype used to annotate unlabeled data examples.

## Domain-Specific Annotations: Use Cases

In this section, we demonstrate the utility of the annotation schema we introduced in the previous section on two use cases, i.e., annotation of datasets for the domains of neurodegenerative diseases and Earth Observation (EO). For the two use cases, we also enriched the annotation schema with terminology specific to the domain at hand. The inclusion of domain-specific annotations improves the representation of the datasets, making them accessible and reusable, offers the possibility of execution of advanced query scenarios, and enables interoperability with other data from the domain.

On a technical level, the alignment of the DM-specific annotation schema with the annotation schemas designed for the particular domains is straightforward. In that sense, the proposed ontology-based annotation schema enables the direct extension of the datatype classes at any level in the taxonomy with classes that define the semantic meaning of the domain-specific datatypes. The newly introduced datatype classes are then linked to the corresponding entities in the domain ontologies.

### Neurodegenerative Disease Datasets

Neurodegenerative diseases such as Alzheimer’s disease (AD), Parkinson’s disease (PD), amyotrophic lateral sclerosis (ALS), and Huntington’s disease (HD) are a group of diseases caused by a progressive loss of structure or function of neurons. They can lead to irreversible deterioration of cognitive functions like memory loss, cause problems with movement, and spatial orientation. In the past two decades, researchers have been investigating new treatments that can slow or stop the progression of the diseases. There are two widely-known studies concerning neurodegenerative diseases, i.e., Alzheimer’s disease Neuroimaging Initiative (ADNI) [19] and Parkinson’s Progression Markers Initiative (PPMI) [4].

To annotate the datasets with terms relevant to the domain, we use the NDDO (Neurodegenerative Disease Data Ontology) ontology [12]. NDDO is designed in accordance with the ADNI and PPMI studies and it is aligned with the OntoDT and OntoDM-core ontologies. Thus, it can be easily adjusted to annotate the four aspects of data examples we considered in Sect. 3. To illustrate this, we use an instance dataset from the PPMI study that [15] used for the task of predicting the motor impairment assessment scores by utilizing the values of regions of interest (ROIs) from fMRI imaging assessment and DaT scans. The DM task they were solving was multi-target regression (MTR).

**Fig. 7.**
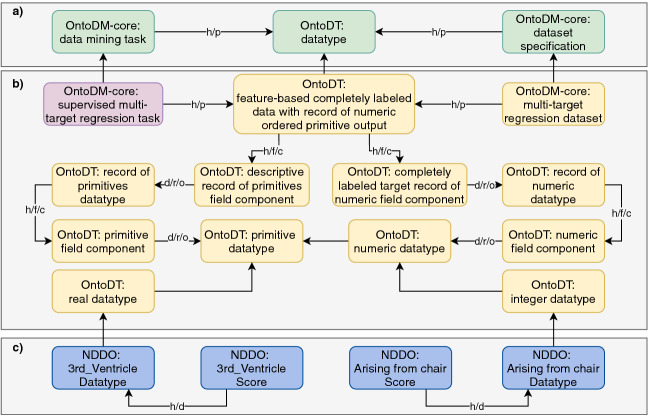
A semantic annotation schema for the PPMI dataset [15]: a) top level classes from OntoDM and OntoDT; b) specific classes and relations required for annotation of datasets used in cluster analysis and c) specific NDDO datatype classes.

Figure 7, depicts the point of alignment of the domain classes defined in NDDO with classes from the extended versions of the OntoDT and OntoDM-core ontologies. To represent the MTR task and MTR dataset specification, we use the classes defined in OntoDM-core, and connect them with the corresponding datatype class from OntoDT (in our case *OntoDT: feature-based completely labeled data with record of numeric ordered primitive output*) (see Fig. 7 b). This class has two field components. The first one describes the datatypes of the descriptive features, which are of a primitive datatype. The latter describes the datatypes of the features on the target side. In the MTR learning setting each target feature is described with the numeric datatype. The sub-classes of the numeric datatype, real and integer datatype, are positioned at the bottom of the datatype taxonomy, and we link them with the domain datatypes.

For example, *NDDO: 3rd_Ventricle Score* is one of the descriptive features present in the PPMI dataset and it is linked with the *NDDO: 3rd_Ventricle Datatype* class that semantically defines its datatype. Similarly, *NDDO: Arising from the chair Score* is a target and its associated datatype is the *NDDO: Arising from the chair Datatype* class. Other features are connected with the respective datatypes in the same way.

### Earth Observation (EO) Datasets

Remote sensing (RS) is the process of monitoring specific physical characteristics of an area of interest by measuring the reflected and emitted energy at a distance from the target area. Satellite-based remote sensing technologies are commonly used for Earth Observation (EO) to monitor characteristics that change over time, i.e., weather prediction, natural changes of the Earth, and development of the urban area.

Due to the increasing availability of EO data, it is essential to develop an ontological approach to managing this kind of data. However, to the best of our knowledge, a general ontology that systematically describes the EO domain is still lacking. Nonetheless, some ontologies formalize the knowledge of specific parts of the domain, i.e., Semantic Sensor Network (SSN) ontology [6], SOSA (Sensor, Observation, Sample, and Actuator) ontology [10], Semantic Web for Earth and Environment Technology (SWEET) ontology [20], and the Extensible Observation Ontology (OBOE) [14].

For semantic annotation of EO data, we have designed a lightweight ontology that is aligned with the aforementioned EO ontologies. The ontology is available at https://w3id.org/eo-ontology. The ontology was constructed using the bottom-up approach, based on 4 instances of datasets we have available at our side from previous research [22].

The datasets contain two target features (forest vegetation height and canopy cover) whose values are obtained via the LiDAR technology. But since LiDAR can sometimes be inconvenient or expensive, [22] examined the possibility of using remote sensing data generated from satellites, such as Landsat 7, IRS-P6, SPOT, as well as aerial photographs for the construction of descriptive features that can be relevant for the prediction of the two targets. The Landsat 7, IRS-P6, and SPOT satellites use multiple channels for collecting reflected energy, and one channel of emitted energy, that operate on different wavelengths.

**Fig. 8.**
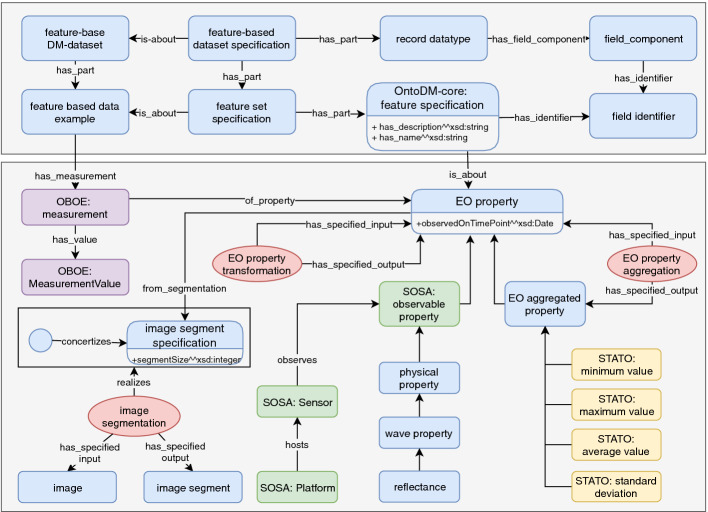
Core entities of the Earth Observations ontology. Rectangular boxes represent continuant classes, while ellipses represent process classes. The color scheme was chosen for better visual perception.

In this study, when designing the EO ontology, we took into consideration the process of data collection and data preprocessing described in the study mentioned above. In the preprocessing phase, the raw satellite image is converted into a standard geo-referenced data format, which then undergoes the process of image segmentation (see Fig. 8). A key characteristic of the different image segments is the resolution of the segment size. The image segment size is modeled as a data property of the image segmentation specification class.

All features present in the datasets are EO properties observed at a specific point in time, and they are related to a specific image segment. We define two subclasses of the EO property class, i.e., *SOSA: observable property* class and *EO aggregated property* class. The first one refers to the properties observed with a remote sensor (*SOSA: Sensor*) hosted on a given platform/satellite (*SOSA: Platform*). The latter defines the type of properties that are the result of some process of EO property aggregation that transforms the originally observed measurements. The process uses multiple EO properties as input and produces one EO aggregated property. The aggregation can be based on some statistical characteristics, such as *STATO: minimum value*, *STATO: maximum value*, *STATO: average value* and *STATO: standard deviation*, where STATO is an ontology of statistical methods. This was also the case in our observed datasets. Additionally, we define the EO property transformation process that transforms one EO property into another.

Similarly, as in Sect. 4.1, to achieve full interoperability, we integrated the general DM annotations with the domain-specific ones. The integration was perforemed at the level of features appearing in the dataset. Thus, the *OntoDM-core: feature specification* class connects with the datatype of the feature via the has-identifier relation, while it also connects with the EO property class via the is-about relation. Additionally, the *OntoDM-core: feature-based data example* class is composed of multiple *OBOE: Measurements*. In OBOE, measurement represents a measurable characteristic of an observed property, which in our case is *EO property*.

## Conclusions and Future Work

We have developed an ontology-based annotation schema for rich semantic annotation of DM datasets that takes into consideration 3 different semantic aspects of the datasets: provenance, DM-specific characteristics of the data, and domain-specific information. The annotation schema is generic enough to support the easy extension of its core classes with information relevant to the application domain. The utility of the designed schema was demonstrated through semantic annotation of data from two different domains: neurodegenerative diseases and Earth observation.

Annotations based on this schema provide means for support of the complete data analysis process, e.g., enable cross-domain interoperability, assist in the definition of the learning task, ensure consistent representation of datatypes, assess the soundness of data, and automatically reason over the obtained results. These annotations also enable the development of applications that require advanced data querying capabilities. They also enable the development of data repositories that adhere to the highest standards of the Open data initiative.

## Footnotes

1 https://json-ld.org/.

2 Fuseki dataset containing the semantic annotations in RDF format: http://semantichub.ijs.si/fuseki/dataset.html?tab=query&ds=/DMDatasets.
